# Thrombotic Thrombocytopenic Purpura Associated with Dermatomyositis

**DOI:** 10.7759/cureus.3161

**Published:** 2018-08-20

**Authors:** Zohra R Malik, Amir Shahbaz, Kashif Aziz, Zareen Razaq, Muhammad Umair, Issac Sachmechi

**Affiliations:** 1 Internal Medicine, Icahn School of Medicine at Mount Sinai/Queens Hospital Center, New York City, USA; 2 Internal Medicine, Icahn School of Medicine at Mount Sinai/Queens Hospital Center, New York, USA; 3 Internal Medicine, Icahn School of Medicine at Mount Sinai Queens Hospital Center, New York, USA; 4 Internal Medicine, Postgraduate Trainee, Ghurki Trust Hospital, Lahore Medical & Dental College, Lahore, PAK; 5 Internal Medicine, Icahn School of Medicine at Mount Sinai Queen Hospital Center, West Hempstead, USA

**Keywords:** dermatomyositis, thrombotic thrombocytopenic purpura (ttp), plasmapheresis

## Abstract

Dermatomyositis and thrombotic thrombocytopenic purpura (TTP) are both rare diseases. TTP is a blood abnormality in which blood clots form in blood vessels leading to fatal outcomes. Dermatomyositis is an inflammatory myopathy which causes a distinctive skin rash and muscle weakness. We are hereby presenting the case of a 27-year-old female who presented with characteristic skin findings on the face pathognomic of dermatomyositis and further investigation revealed that she had underlying TTP.

## Introduction

Dermatomyositis is an idiopathic inflammatory myopathy that is characterized by the features of proximal skeletal muscle weakness and by evidence of muscle inflammation [1]. Dermatomyositis is associated with a variety of characteristic skin manifestations [2]. Thrombotic thrombocytopenic purpura (TTP) is a thrombotic microangiopathy caused by the severely reduced activity of the von Willebrand factor-cleaving protease ADAMTS13. It is characterized by small-vessel platelet-rich thrombi that cause thrombocytopenia and microangiopathic hemolytic anemia (MAHA). Some patients may have neurologic abnormalities, mild renal insufficiency, and low-grade fever. Most cases of TTP are acquired, caused by autoantibody inhibition of ADAMTS13 activity. Hereditary TTP, caused by ADAMTS13 gene-mutations, is much less common [3].

## Case presentation

**Figure 1:**
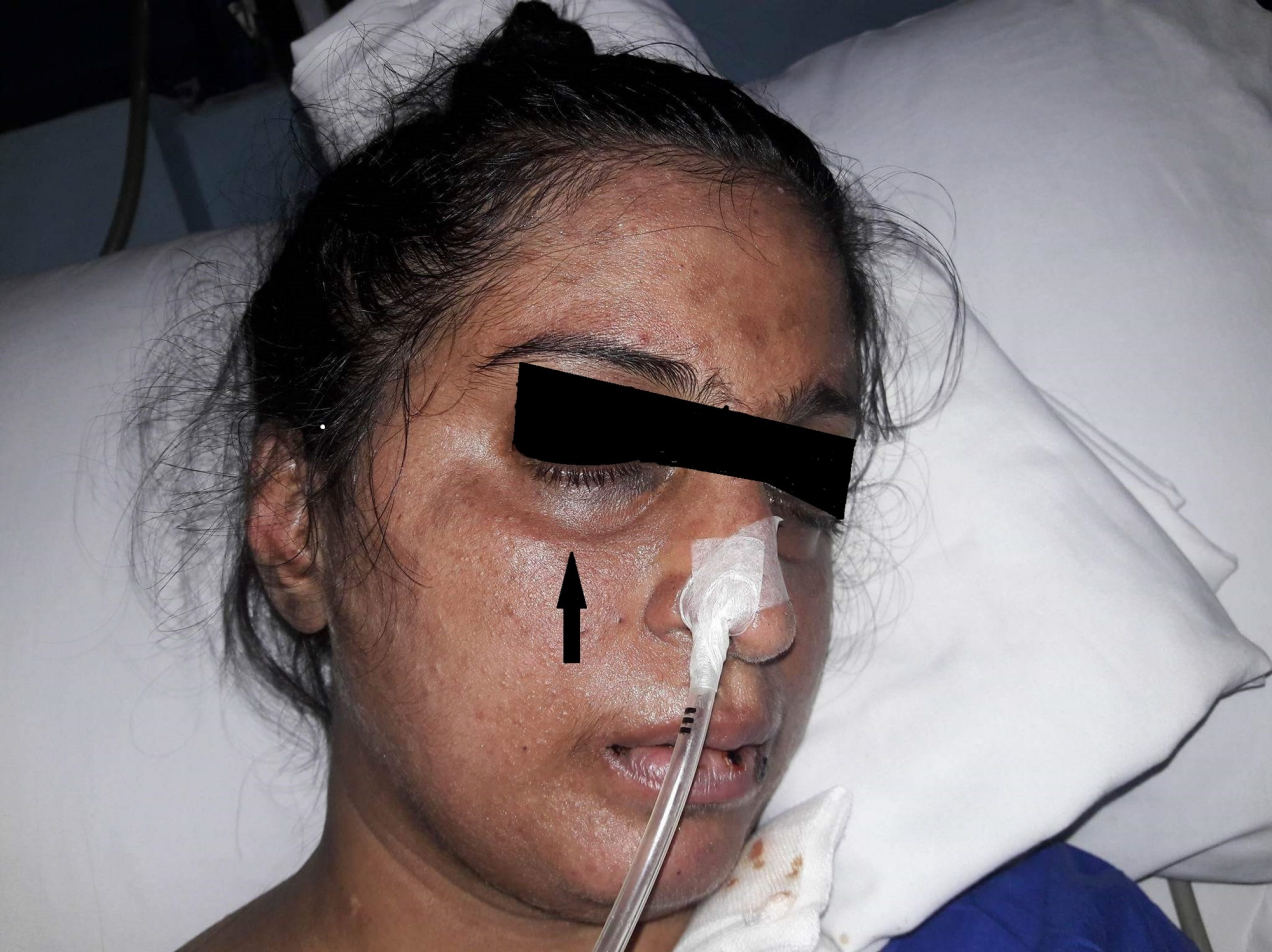
Heliotrope Rash

A 27-year-old female with no past medical or surgical history was admitted with complaints of fever, altered consciousness and hypotension. She reported a rash on the face that has been there for the last one year which did not respond to topical treatment with steroids. She did not have any history of contact with sick people and had not traveled recently. Physical examination revealed typical physical signs of dermatomyositis i.e. heliotrope rash as shown in Figure 1.

Lab work up showed hemoglobin concentration 8.5 g/dl (normal = 12.3-15.5 g/dl), hematocrit 0.28 (normal 0.35-0.44), red blood cells 3.30x10^12^/L (normal 4.2-5.2x10^12^/L, platelet count 40,000/ul (normal = 150,000-450,000/ul), lactate dehydrogenase (LDH) 814 IU/L(normal < 200 IU/L), total bilirubin was 2.2mg/dl (normal = 0.1-1.2mg/dl), prothrombin time (PT) 16 sec (normal = 12-14 sec), activated partial thromboplastin time (aPTT) 38 sec (normal < 35 sec), blood urea nitrogen (BUN) 42 mg/dL (normal 7-20 mg/dl), creatinine 3.5 mg/dL (normal 0.5-1.1 mg/dl). Spinal tap, urinalysis and blood cultures were negative, ruling out any infectious etiology of presentation. A diagnosis of TTP was made owing to presence of fever, altered state of consciousness, renal failure, anemia and thrombocytopenia. Antinuclear antibody (ANA) and anti Jo were positive consistent with autoimmune etiology of dermatomyositis. Patient was treated with plasmapheresis and her condition improved.

## Discussion

The diagnosis of TTP in our case was made based on clinical and laboratory findings of fever, hemolytic anemia, thrombocytopenia, neurological symptoms and renal failure. Although TTP usually occurs without other underlying diseases, some cases have been reported in association with a variety of conditions such as pregnancy, infections, toxins and autoimmune disorders [4]. In order to diagnose TTP in the acute phase of the disease, it is not essential to assay ADAMTS13 [5]. After having ruled out other thrombotic microangiopathies, patients can still be appropriately diagnosed with TTP without the ADAMTS13 assay. There is no effective therapy for TTP, but plasma therapy (plasma exchange, plasmapheresis or infusion), alone or combined with other forms of therapy, can dramatically improve the prognosis of patients with TTP, although the mechanism by which the therapy works is not well understood [6]. The decision to implement plasma therapy (infusion in patients with an inherited disease, exchange in acquired disease) does not warrant the availability of ADAMTS13 values in real time [5]. Other forms of therapy are corticosteroids, antiplatelet agents, high doses of immunoglobulin and vincristine [7-10]. A splenectomy is a treatment option of last resort [11]. As per our patient, there seems to be an association between dermatomyositis and TTP. In the review of the literature, we found only three cases of dermatomyositis complicated by TTP [12-14]. Early diagnosis and prompt treatment with plasmapheresis may improve the outcome of TTP patients with dermatomyositis [6]. Physicians should keep in mind that TTP occasionally arises as a serious complication of dermatomyositis and anyone presenting with a heliotrope rash and symptoms suggestive of TTP should be further investigated, as prompt treatment is the key to survival.

## Conclusions

The possibility of an association of TTP with dermatomyositis should always be considered especially in a person presenting with characteristic skin finding of heliotrope rash, fever, altered levels of consciousness, renal function abnormalities, anemia and thrombocytopenia.
